# Targeting bacterial biofilm-related genes with nanoparticle-based strategies

**DOI:** 10.3389/fmicb.2024.1387114

**Published:** 2024-05-22

**Authors:** Shima Afrasiabi, Alireza Partoazar

**Affiliations:** ^1^Laser Research Center of Dentistry, Dentistry Research Institute, Tehran University of Medical Sciences, Tehran, Iran; ^2^Experimental Medicine Research Center, Tehran University of Medical Sciences, Tehran, Iran

**Keywords:** antibiotic resistance, bacterial pathogens, biofilms, efflux pumps, gene expression, quorum quenching, quorum-sensing, nanoparticles

## Abstract

Persistent infection caused by biofilm is an urgent in medicine that should be tackled by new alternative strategies. Low efficiency of classical treatments and antibiotic resistance are the main concerns of the persistent infection due to biofilm formation which increases the risk of morbidity and mortality. The gene expression patterns in biofilm cells differed from those in planktonic cells. One of the promising approaches against biofilms is nanoparticle (NP)-based therapy in which NPs with multiple mechanisms hinder the resistance of bacterial cells in planktonic or biofilm forms. For instance, NPs such as silver (Ag), zinc oxide (ZnO), titanium dioxide (TiO_2_), copper oxide (Cu), and iron oxide (Fe_3_O_4_) through the different strategies interfere with gene expression of bacteria associated with biofilm. The NPs can penetrate into the biofilm structure and affect the expression of efflux pump, quorum-sensing, and adhesion-related genes, which lead to inhibit the biofilm formation or development. Therefore, understanding and targeting of the genes and molecular basis of bacterial biofilm by NPs point to therapeutic targets that make possible control of biofilm infections. In parallel, the possible impact of NPs on the environment and their cytotoxicity should be avoided through controlled exposure and safety assessments. This study focuses on the biofilm-related genes that are potential targets for the inhibition of bacterial biofilms with highly effective NPs, especially metal or metal oxide NPs.

## 1 Introduction

Approximately 40 to 80% of bacteria possess the ability to create biofilms under harsh environmental conditions. This phenomenon results in a major global concern for human health, as it greatly contributes to the development of persistent infections (Muhammad et al., 2020). Infections caused by biofilms are highly resistant to antibiotics and host immune cells compared to planktonic cells. Unlike planktonic bacteria, biofilms are resistant to conventional cleaning, washing, heating, and disinfection procedures (Sharma et al., 2019). The main reasons for antibiotic resistance of biofilms are the restriction of antibiotic entry to the biofilm structure, slow growth and metabolism, and resistance genes. Biofilm formation on medical implants such as sutures, catheters, heart valves, joint prostheses, and dental implants leads to chronic infections such as wound infections, urinary tract infections, lung infections, and chronic osteomyelitis. Infections caused by implanted medical devices account for about 60–70% of all nosocomial infections. The only way to eliminate biofilms from medical implants is the implant removal which is an expensive and problematic treatment for patients (Cangui-Panchi et al., 2022). Biofilms are homogeneous or heterogeneous microbial communities of microorganisms that live on abiotic or biotic surfaces and consist of cells, extracellular matrix (ECM), and other polymeric substances. EMC is a biopolymer matrix consisting of exopolysaccharides (EPS), extracellular DNA, proteins, and amyloidogenic proteins (Gupta et al., 2016).

There are important genes associated with bacteria that grow in biofilm, including *rhlI*-*rhlR*, *rhlAB*, *pqsR*-*pqsA*, *lasR*, *lecA*, and *pel A* in *Pseudomonas aeruginosa* (Xu et al., 2018; Abdelraheem et al., 2020), while *icaADBC*, *eno* (laminin), *ebps* (elastin), *fib* (fibrinogen-binding protein) are necessary for biofilm formation in *Staphylococcus aureus* (Kot et al., 2018). The *gtfs* genes are essential for the adhesion of *Streptococcus mutans*. In addition, the genes *gbps*, *smu630*, *relA*, and *comDE* are involved in bacterial adhesion and biofilm formation (You, 2019). Type I fimbriae encoded by the gene cluster *fimABCDEFGH* are involved in the attachment of *Escherichia coli* cells (Ren et al., 2004).

Some of the important strategies being developed against biofilms are quorum sensing inhibitors (QSIs), bacteriophages, enzymes, surfactants, nanoparticles (NPs), antimicrobial photodynamic therapy, ethnopharmacology, and diguanylate cyclase inhibitors (Qayyum and Khan, 2016). Although numerous antimicrobial drugs are commercially available, they often lack efficacy against multidrug-resistant (MDR) microorganisms, posing a major challenge to healthcare teams (Ahmed et al., 2021). The emergence of antimicrobial resistance has increased the need for the development of alternate antimicrobial agents (Uddin et al., 2021).

Nanotechnology is a promising tool for the management of MDR strains and bacterial biofilms. In recent years, the applications of NPs in the medical field have increased for the treatment of infectious diseases. NPs possess different physicochemical properties than bulk forms due to their unique size and structure at the nanoscale. In the size range of 1 nm to 100 nm, NPs exhibit enhanced surface-to-volume ratio, increased reactivity, altered electronic properties, etc., depending on size (Baig et al., 2021). The ability of NPs to inhibit the proliferation of microbial cells makes them the most widely used drug delivery system to combat pathogenic organisms (Lahiri et al., 2021).

The unique properties of metal NPs have led to their great interest as antimicrobial agents. The antimicrobial properties of various metal NPs, including silver (Ag), zinc oxide (ZnO), titanium dioxide (TiO_2_), copper (Cu), iron oxide (Fe_3_O_4_), selenium (Se), and gold (Au) have been extensively investigated (Hemeg, 2017). Previous research suggests that NPs have different mechanisms of action than antibiotics, which may improve their ability to combat MDR bacteria. Antibacterial activity studies have shown that NPs can enter the cell and attach to cell receptors, leading to intracellular disintegration and consequent cell death, and inhibit essential metabolic enzymes, thereby disrupting bacterial cell reproduction and respiration. NPs have been developed that interfere with signal-based biofilm formation to prevent biofilm-associated infections (Gupta et al., 2017; Wang et al., 2017). The review describes the efficacy of highly potent NPs, especially metal or metal oxide NPs, as potent anti-biofilm therapeutics with particular reference to their ability to inhibit bacterial biofilm-related genes.

## 2 Biofilm formation process

**Figure 1:**
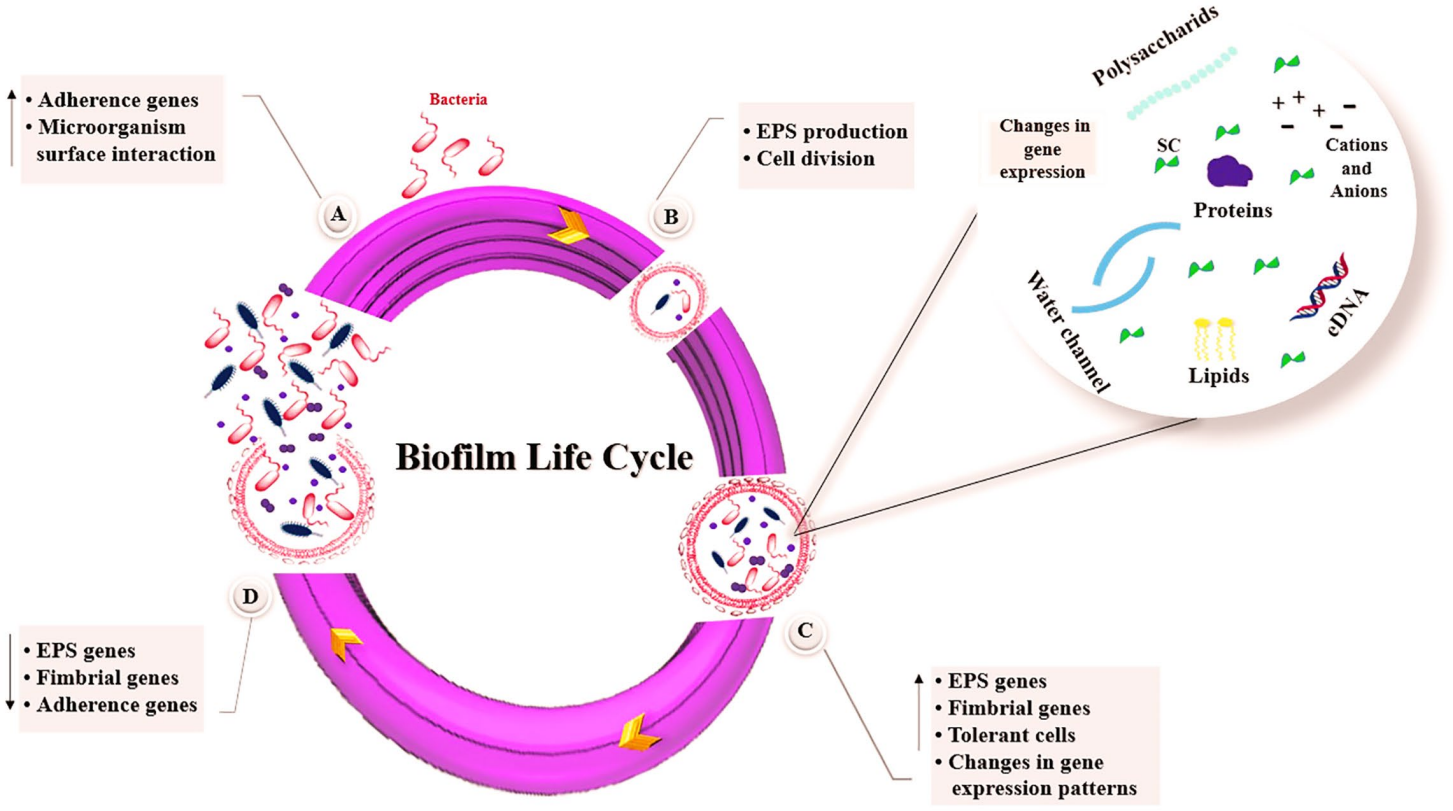
Stages of biofilm formation. **(A)** Bacteria attachment, **(B)** Microcolony formation, **(C)** Biofilm maturation, **(D)** Biofilm dispersal. SC, signaling compounds; eDNA, extracellular DNA; EPS, exopolysaccharides.

Biofilm formation is a survival strategy of microorganisms in which microbial cells adapt to their environment and adopt a multicellular lifestyle in which bacterial cells are immobilized within their EPS matrix. The bacteria within this matrix are protected from antibacterial compounds and are up to 1,000 times more resistant to antibiotics (Uneputty et al., 2022). Biofilm formation begins with reversible attachment of free-floating bacterial cells to a surface. This is followed by irreversible attachment, which is facilitated by bacterial adhesion structures and short-range forces. The process of EPS production drives reversible attachment to the surface. They then develop into a well-organized structure encased in an EPS matrix. Ultimately, the bacterial cells are able to escape and access new niches (Muhammad et al., 2020). The stages of biofilm life cycle are depicted in Figure 1. Five key stages of biofilm formation are presented below.

### 2.1 Bacterial initial attachment and irreversible adhesion

Bacterial attachment to surfaces favors in biofilm formation in initial step. Initially, the bacterium gets close enough to allow initial attachment, and the forces involved in this initial attachment are van der Waals forces, electrostatic and hydrophobic interactions. During this initial contact, the bacteria still exhibit Brownian motion and can be easily removed by the flow rate of the liquid. Subsequently, an irreversible step occurs, as the bacteria attach to the surface by producing EPS and or filamentous appendages, such as pili or flagella and non-fimbrial adhesions. At the end of this phase, much stronger physical or chemical forces are needed to remove the bacteria attached to the surface (Palmer et al., 2007). The size of the contact area between bacterial cell and material surface, the total of the repulsive or attractive forces between two surfaces, the hydrophobicity/hydrophilicity, roughness and surface charge, the physicochemical and topographic properties of a substrate surface can all influence the attachment of bacteria (Renner and Weibel, 2011). In *S. aureus*, *atlE* gene expression mediates bacterial adhesion and secretes autolysin. Strains lacking the *atlE* gene exhibit a marked reduction in adhesion capacity. The expression of *fbe* and *sap* genes leads to synthesis of fiber protein binding protein, which mediates the attachment of *S. aureus* and fibrin. During the accumulation phase, *iapABD* gene is expressed to synthesize the ECM and eventually form pileus-like multilayer structure in mature biofilms (Zhao and Ashraf, 2015).

### 2.2 EPS synthesis

Irreversible adhesion is promoted by the synthesis of EPS, which is controlled by the QS of the bacterial cells. Bacteria produce EPS, which is an important element of biofilm ECM. EPS can mediate bacterial adhesion–cohesion via hydrophobic and ion-bridging forces. Overall, EPS directly mediates adherence to microbial cell surfaces, cell-to-cell adhesion, biofilm structure, biofilm formation, water retention, cell–cell signaling, cell protection, nutrient trapping capability, and genetic adaptation. In addition, the cyclic messenger di-GMP (c-di-GMP) is considered to be one of the triggers for the switching of reversible to irreversible attachment via EPS and cell surface structures. EPS are mainly enzymes and structural proteins, DNAs, polysaccharides, phospholipids, and glycoproteins (Czaczyk and Myszka, 2007). Polysaccharides are a key fraction of the EPS matrix and are necessary during the biofilm development process and growth of the bacteria (Flemming, 2016).

### 2.3 Microcolony formation

Microbial cells begin to multiply and divide after they connect to a biotic or abiotic surface and this attachment becomes stable. This process is started by specific chemical signaling within the EPS. The next step in this process is the formation of microcolonies. The different micro-communities that make up the bacterial colonies in a biofilm usually coordinate with each other in different ways. The exchange of substrate, the distribution of important metabolic products, and the excretion of metabolic end products all depend on this coordination (Gestel et al., 2015).

### 2.4 Biofilm maturation

The EPS-embedded bacteria conduct to microcolonies formation and biofilms maturation. The EPS then acts as a biological ‘glue’ between the embedded bacterial cells through changes in gene expression. Through matrix formation, nutrients are transported to the cell communities and unwanted products are removed through the formation of water-filled channels. Often a mushroom-shaped multicellular structure of microcolonies is displayed. During the maturation process, inhibition of the production of surface structures restricts motility in microcolonies and the pattern of gene expression differs significantly from that of planktonic cells (Muhammad et al., 2020).

### 2.5 Biofilm dispersal

The dispersal process is a tactic used by bacterial cells to breakdown biofilms and begin a new biofilm life-cycle. The complicated process of dispersal is controlled by effectors, signal transduction pathways, and environmental signals. Genes responsible for the EPS generation, initial attachment, and fimbriae synthesis are often reduced during dispersal, but genes related to EPS degradation and flagella synthesis are typically enhanced. Inhibition of c-di-GMP signaling pathways is another effective method of biofilm dispersion. Furthermore, environmental factors (i.e., temperature, nutrients, and pH) increase in glucose supply, and lack of oxygen can contribute to biofilm dispersal (Toyofuku et al., 2016).

## 3 QS mechanism

QS is a chemical communication mechanism that correlates with population density and is used by bacteria to regulate the production of virulence factors and biofilm formation. The elimination of QS is a new approach to combat their pathogenicity (Colino et al., 2018). They implement QS by secreting small extracellular signaling molecules that act as autoinducers (AI) to start genetic programming. Three main QS systems can be distinguished: the acyl-homoserine lactone (AHL) QS system in Gram-negative bacteria, the autoinducing peptide (AIP) QS system in Gram positive bacteria, and the AI-2 QS system in both Gram-negative and Gram-positive bacteria. QS is associated with the control of the swarming behavior of bacteria and the development of biofilm architecture (Sadekuzzaman et al., 2015).

Quorum quenching (QQ) refers to any strategy that interferes with proper microbial QS signaling. This can occur in two ways: inhibition and degradation of AI and disruption of its interaction with the receptor. These compounds have been characterized as QQ, which interfere with bacterial communication, reducing virulence without affecting growth. Depending on the type of regulation (i.e., whether QS induces or suppresses virulence), agents must either inhibit or stimulate QS-regulated gene expression (Colino et al., 2018). QQ enzymes have frequently been found to inhibit QS by targeting AHLs (Hong et al., 2012).

## 4 Efflux pumps

Efflux pumps are membrane proteins that are involved in the development of antibiotic resistance and the export of molecules such as QS signals, drugs, detergents, and heavy metals outside of the cell. Efflux pump genes are present in bacterial chromosomes and mobile genetic elements such as plasmids (Webber and Piddock, 2003). At least four different roles for efflux pumps in biofilm formation are possible. Efflux of EPSs and/or QS and QQ molecules to facilitate biofilm matrix formation and regulate QS, respectively; they could also indirectly regulate genes involved in biofilm formation; efflux of harmful molecules, such as antibiotics and metabolic intermediates; and influence aggregation by inhibiting adhesion to surfaces and other cells (Alav et al., 2018). In *E. coli* biofilm development, the efflux genes *araJ*, *ddpD*, *emrK*, *gltK*, *ycbO*, and *yhdX* increased during biofilm growth (May et al., 2009). Sánchez et al. reported that *P. aeruginosa nalB* and *nfxB* mutants, which overexpress MexAB-OprM and MexCD-OprJ efflux systems, respectively, did not show any defects in biofilm formation and, in fact, the *nalB* mutant strains were found to exhibit significantly denser biofilm formation when compared to the wild-type (Sánchez et al., 2002). One possibility is that the overexpression of multidrug efflux pump MexEF-OprN reduces the intracellular concentration of QS signals exhibited impaired biofilm formation (Alav et al., 2018). Holling et al. reported that disruption of the *bcr* efflux gene in *Proteus mirabilis* led to reduced biofilm formation. Furthermore, the *bcr* mutant exhibited deficiencies in both swarming and swimming motility (Holling et al., 2014).

## 5 Intercellular adhesion operon

The intercellular adhesion (ica) operon, which includes the genes *icaA*, *icaD*, *icaB*, and *icaC*, encodes biosynthetic enzymes involved in the synthesis of polysaccharide intercellular adhesin (PIA). EPS synthesis is closely linked to the *icaA* and *icaD* genes. Similarly, *icaB* and *icaC* contribute to the production of poly-N-acetylglucosamine polymer by transporting it to the bacterial cell surface and deacetylating exopolysaccharide molecules. The N-acetylglucosaminyltransferase acts on the substrate UDP-N-acetylglucosamine with the help of *icaD* and forms an EPS. The reaction product is then transported across the cytoplasmic membrane by *icaC*. Deacetylation of PIA by *icaB* in turn enables PIA to adhere to living and non-living surfaces and facilitates biofilm formation. Although the PIA adhesin plays a key role in the process of biofilm synthesis, it is not essential for biofilm production. There are biofilm-forming strains that do not contain *ica* operon genes (Swolana et al., 2022).

## 6 Strategies for prevention of biofilm formation

Biofilm formation is a complicated process that can be targeted by anti-biofilm agents to prevent the various stages of biofilm development (Mahamuni-Badiger et al., 2020). Several genes are involved in biofilm formation, so actions can be taken at the genetic level, such as interfering with second messenger signals and the two-component system (TCS), to modulate EPS metabolism (Karatan and Watnick, 2009). C-di-AMP and c-di-GMP are conserved second messenger signals that are crucial for important functions of virulence, such as biofilm formation. They control various EPS-producing exoenzymes, polysaccharides, and adhesins. It is therefore possible to target them to disrupt EPS (Jiang et al., 2020). Antisense RNA is complementary to messenger RNA and its duplex can interfere with the translation of specific proteins (Yoshioka et al., 2019). Antisense RNA could manipulate the expression of target genes and control the biofilm (Wang and Kuramitsu, 2005). Bacteria have an adaptive immune defense mechanism known as the CRISPR/Cas system (clustered regularly interspaced short palindromic repeats). Biofilm formation, EPS synthesis, interspecific competitiveness, and other aspects of bacterial virulence are influenced by the CRISPR/Cas system (Arora, 2024). The CRISPR/Cas9 system targeting *sdiA* has been reported to have effects on cell adhesion and biofilm formation of *Salmonella enterica* (Askoura et al., 2021). Furthermore, the CRISPR/Cas-HDR approach was used to prevent biofilm formation of *E. coli* by knockout genes involved in adhesion (*fimH*/*bolA*) and QS (*luxS*; Alshammari et al., 2023). On the other hand, the expression of *wcaF*, which is involved in colanic acid synthesis and has been identified as a critical component of the *E. coli* biofilm, was controlled using the CRISPRi/dCas9 system (Zhang and Poh, 2018).

Biofilm formation can be prevented by avoiding the initial attachment of planktonic cells to surfaces by remodeling the surface or treating the cells to block cell attachment. Preformed biofilms can be removed by detachment, erosion, and dispersal (Mahamuni-Badiger et al., 2020). Furthermore, anti-biofilm agents can act in different ways by inhibiting or altering QS signaling pathways, membrane permeabilization, biofilm assembly, lipopolysaccharide assembly, and enzymatic dispersion of EPS (Roy et al., 2018; Asma et al., 2022).

## 7 NPs as inhibitors of biofilm formation

Nanotechnology approaches have great potential to offer effective and practical solutions for biofilm management and prevention, as they deal with the engineering of materials at the atomic and molecular level that have high surface-to-volume ratios and high activation energy of atoms (Mohanta et al., 2023). It is a good platform to generate effective antimicrobial agents by optimizing the physicochemical properties of metals. Metal oxides are toxic and harmful to natural sources, but at the nanoscale they change their physicochemical properties. The ability of NPs to penetrate biofilms also inhibits biofilm formation (Mohanta et al., 2023). Three stages can be distinguished in the interaction between NPs and biofilm: NP transport in the vicinity of the biofilm, NP attachment to the biofilm surface, and NP migration in biofilms. Many factors, including the environment, EPM, and the physico-chemical properties of the NPs, influence how the individual phases take place (Shkodenko et al., 2020).

**Figure 2:**
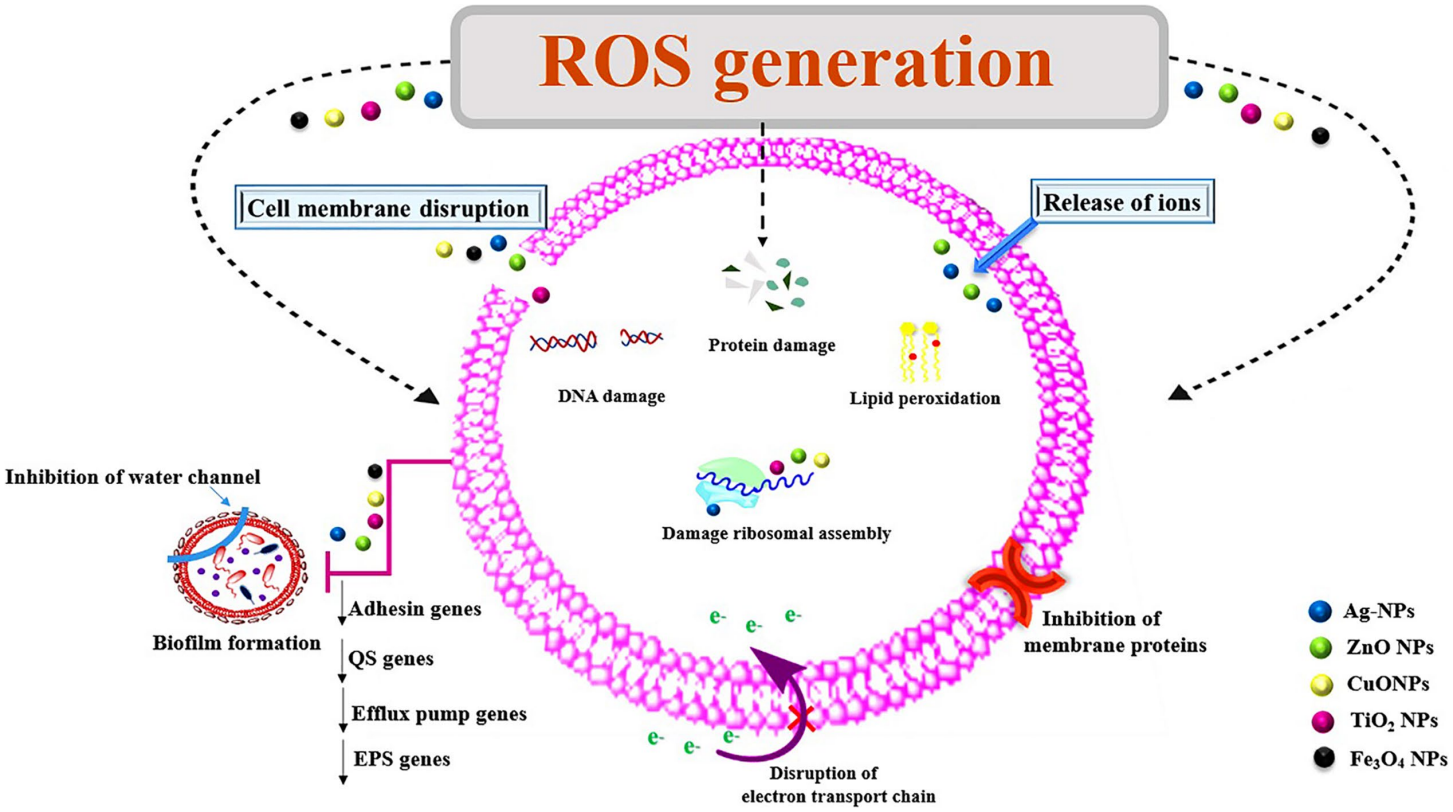
Mechanisms of action of nanoparticles for their antimicrobial properties. ROS, reactive oxygen species; NPs, nanoparticles; Ag, silver; ZnO, zinc oxide; TiO_2_, titanium dioxide; Cu, copper oxide; Fe_3_O_4_, iron oxide; EPS, exopolysaccharides; QS, quorum sensing.

Some NPs themselves have the ability to demonstrate anti-biofilm activities because they contain antibacterial components such metal oxides and cationic surfactants (Sadekuzzaman et al., 2015). Their large surface area to mass ratio, high reactivity, and surface functionalization have given them special qualities that enable effective biofilm eradication. NPs act as anti-QS by interfering with the mechanism of bacterial cell–cell communication or inhibiting the QS signaling system. This is able to prevent production of molecule-receptor complex and the formation of various signaling molecules. The abilities of metallic NPs to exert QQ activity have been particularly noted (Lahiri et al., 2021). As given in Figure 2, NPs can exert their antibacterial activity through different mechanisms, such as cell wall disruption, interactions with DNA and/or proteins, inhibition of biofilm development, or generation of reactive oxygen species (ROS; Wang et al., 2017).

### 7.1 Ag NPs

Ag NPs are widely used in healthcare and biomedicine, food storage, and environmental applications. Ag NPs are remarkable for their unique physicochemical features such as excellent catalytic activity and stability, high conductivity, and significant anti-inflammatory and antibacterial activities (Lee and Jun, 2019). Interference with cell-membrane function and intracellular ROS generation have been the key pathways for antibacterial activity (Liao et al., 2019).

Ag NPs probably destabilize the cell membrane and produce Ag ions that react with membrane proteins. These NPs have the ability to pass through bacterial cell walls and/or membranes, attach to bacterial DNA, and obstruct DNA replication. Furthermore, they have the ability to disrupt ribosomal function to translate mRNA into protein forms, thereby activating cytochrome B proteins (Yan et al., 2018). The antibacterial mechanism of Ag NPs works by inhibiting O_2_ metabolism, eventually killing microorganisms (Shahverdi et al., 2007). Furthermore, Ag NPs can efficiently diminish bacterial biofilm biomass (Montazeri et al., 2020).

The anti-biofilm activity and downregulation of bacterial biofilm-related genes of Ag NPs against a variety of bacteria has been reported in several research papers (Table 1). Ag NPs have the ability to block the synthesis of bacterial EPS and then biofilm. The ability of biofilm inhibition by Ag NPs may be due to the existence of water channels throughout the biofilm (Kalishwaralal et al., 2010; Montazeri et al., 2020; Pourmbarak Mahnaie and Mahmoudi, 2020). They can enter biofilms and inhibit biofilm development by suppressing gene expression. These ions are internalized and prevent the penetration of amines, thiols, or carboxylates. Ag NPs tend to agglomerate, which negatively affects their antimicrobial efficacy. Therefore, their surface must be functionalized before application (Le Ouay and Stellacci, 2015).

**Table 1:** Summary of the effect of silver nanoparticles and their related products on the downregulation of bacterial biofilm-related genes.

Compound name	Target microorganisms	Target genes	Applications	References
D-SNPs- AgNO_3_	MRSA	*hly*, *flu*	Anti-biofilm effect	Hamida et al. (2020)
Ag NP	*Pseudomonas putida*	*csgA*, *alg8*	EPS inhibition	Thuptimdang et al. (2015)
PbAgNP	*Serratia marcescens* *Proteus mirabilis*	*fimA*, *fimC*, *flhD*, *bsmB* *flhB*, *flhD*, *rsbA*	Anti-biofilm effect-QSI	Srinivasan et al. (2018)
Ag@Glu/TscNP	MRSA *Pseudomonas aeruginosa*	*icaA*, *icaD* *pslA*	Anti-biofilm effect	Montazeri et al. (2020); Honarmand et al. (2022)
AMP@PDA@AgNP	*P. aeruginosa* *Escherichia coli*	*rh II*, *las I* *fim H*	Anti-biofilm effect	Xu et al. (2021)
Ka-AgNPs	*P. aeruginosa*	*lasA, lasB, lasI, rhlA, rhlB, rhlR, rhlI, pqsC*	Anti-biofilm effect	Kumar et al. (2022)
Phytosynthesized AgNP	MDR-*Klebsiella pneumoniae*	*mrkA, luxS*	Anti-biofilm effect-QSI	Foroohimanjili et al. (2020)
Ag NP–4NPO	*P. aeruginosa*	*rhlA*	Anti-biofilm effect	Liu et al. (2019)
GO-Ag NP	*Streptococcus mutans* *Porphyromonas gingivalis*	*gtfB*, *gtfC*, *gtfD* *FimA*	Anti-biofilm effect	Jin et al. (2017)
Ag/ZnO	*S. mutans*	*spaP*, *gbpB*, *gtfB*, *gtfC*, *ldh*, *comD*, *comE*, *luxS*	Anti-biofilm effect	Huang et al. (2020)
AgNPs @C	*P. aeruginosa*	*fimH*, *rmpA*, *mrkA*	Anti-biofilm effect	Elwakil et al. (2024)
QADM- Ag NP	*S. mutans*	*gtfB*, *gtfC*, *gtfD*	Anti-biofilm effect	Li et al. (2013)
AgNP@A	*Staphylococcus aureus*	*icaA*, *icaR*	Anti-biofilm effect	Moulavi et al. (2019)
TzAgNP	*P. aeruginosa*	*lasI*	Anti-biofilm effect	Chakraborty et al. (2021)
CNPs FNPs	*P. aeruginosa*	*lecA*, *lecB*, *lasI*, *pslA* *lecA*, *lasI*, *rhlI*, *pslA*	Anti-biofilm effect	Bhargava et al. (2018)
Ag NP	*Staphylococcus epidermidis*	*icaA*, *icaB*, *icaC*, *icaR*	Anti-biofilm effect	Swolana et al. (2022)

D-SNPs-AgNO_3_, silver nanoparticles (Ag NPs) synthesized using Desertifilum sp.-silver nitrate; MRSA, methicillin-resistant *Staphylococcus aureus*; PbAgNP, Piper betle-based synthesized Ag NPs; Ag@Glu/Tsc NP, Thiosemicarbazide conjugated with Ag NPs and functionalized by glutamic acid; AMP@PDA@AgNP, Antibacterial peptides- polydopamine@Ag NPs; MDR, multidrug-resistant; Ag NP–4NPO, Ag NP combined with the quorum sensing inhibitor (QSI) 4-nitropyridine N-oxide; GO, graphene oxide; AgNPs@C, Ag NP coated by a carbon shell; Ka-Ag NPs, Koelreuteria paniculata for the biosynthesis of Ag NPs; QADM, quaternary ammonium dimethacrylate; Ag NP@A, Ag NPs fabricated from Artemisia scoporia extract; TzAgNP, tetrazine-capped Ag NPs; CNPs, citrate capped Ag NPs; FNPs, fucose functionalized Ag NP.

Ag has a strong inhibitory effect on the strong biofilms produced by *Acinetobacter baumannii.* In presence of Ag NPs, the expression of major adhesion virulence factors of *A. baumannii* such as group 2 capsule synthesis (*kpsMII*) and *afa/draBC* adhesin genes decreased by 4.6-folds and 3.4-folds, respectively. Furthermore, in comparison to the control, the transcription of biofilm-related genes (*bap, OmpA,* and *csuA/B*) was drastically reduced by 4.5-, 3.1-, and 3.1-folds, respectively (Hetta et al., 2021).

Miola et al. (2015) assert that Ag NPs mixed with poly methyl methacrylate (PMMA)-based bone cement significantly reduced biofilm formation. The main mechanism of this Ag NP–PMMA is inhibition of bacterial colonization. Ag hydroxyapatite nanocomposite (Ag/HA-NC) can obviously inhibit *S. aureus* biofilm formation. Bacterial adhesion and biofilm formation on the Ag/HA-NC coating were significantly lower than on the HA coating. In addition, the expression of *atlE*, *fbe*, *sap*, and *iapB* genes, all involved in initial attachment of *S. aureus* was inhibited with Ag/HA-NC. Therefore, it can be concluded that the antibacterial effect of Ag/HA-NC is achieved due to the release of Ag NPs, which supports the clinical use of Ag/HA-NC (Zhao and Ashraf, 2015).

Silencing of QS is a novel approach to combat the pathogenicity of *P. aeruginosa*. Mycofabricated Ag NPs with metabolites of the soil fungus *Rhizopus arrhizus* BRS-07 were able to decrease LasIR-RhlIR levels, inhibit biofilm formation, and significantly reduce the expression of QS-regulated genes (*lasI*, *lasR*, *rhlI*, *rhlR*; Singh et al., 2015). In addition, Ag NPs stabilized with glutathione (GSH-Ag NPs) at a concentration of ½ MIC was found to have anti-biofilm activity in *P. aeruginosa* by reducing the expression of *lasR* and *lasI* genes (Pourmbarak Mahnaie and Mahmoudi, 2020). One study found that in resistant *Klebsiella pneumoniae* strains, the expression of the efflux pump gene *OxqAB* was decreased by both commercial and biosynthesized Ag NPs (Dolatabadi et al., 2021). What is more, Ag NPs were potential NPs that showed excellent reduction of biofilm-related genes (*fimH*, *rmpA*, and *mrkA*) in *K. pneumoniae* (Mousavi et al., 2023).

Wang et al., developed the Ag NP-functionalized titanium implant surface. The Ag NP-doped surface reduced the expression of *ica*A gene for *Staphylococcus epidermidis* and *fnb*A and *fnb*B genes for methicillin-resistant *S. aureus* to decrease the production of intercellular polysaccharide adhesin and fibronectin-binding proteins, thereby reducing bacterial adhesion and biofilm formation (Wang et al., 2016). Although Ag NPs have been extensively studied for antibacterial applications, their production and use are subject to many limitations, including the inability to control size and shape, aggregation, colloidal stability, and potential toxicity at high doses (Jena et al., 2012; Zhang et al., 2019).

### 7.2 ZnO NPs

ZnO NPs are widely known for their antibacterial and anti-biofilm activity against a wide range of bacteria such as *P. aeruginosa*, *Streptococcus pneumoniae*, *Listeria monocytogens*, *Salmonella enteritidis*, and *E. coli* with low toxicity to human cells (Mahamuni-Badiger et al., 2020). Thanks to their potent antibacterial action, these NPs can reduce microbial adhesion, proliferation, and biofilm growth (Campoccia et al., 2013). However, their antibacterial activity is influenced by various factors such as UV illumination, size, shape, concentration, surface modifications, and surface defects (Mahamuni-Badiger et al., 2020).

Recently, ZnO NPs have gained even more attention as they possess the highest toxicity against drug resistant microorganisms. ZnO NPs exhibit a different type of antimicrobial action compared to other NPs. They first destroy the bacterial cell wall, then enter the cell, and finally accumulate in the cell membrane, leading to death (Li et al., 2012). These NPs can alter the microenvironment near the bacteria and liberate ROS species and Zn^2+^ ions cause damaging lipids, proteins, carbohydrates, and DNA by oxidative stress, lipid peroxidation, and disrupting vital cellular functions (Sondi and Salopek-Sondi, 2004). Among NPs, they are strongly preferred because ZnO is recognized as a safe material by the US Food and Drug Administration (De Romana et al., 2002). ZnO meets the requirements for an ideal anti-biofilm agent (Hou et al., 2018).

QS inhibitory effect of ZnO NPs has been demonstrated by reducing the production of *P. aeruginosa* virulence factors such as rhamnolipids, pyocyanin, pyoverdin, hemolysins, elastase, and proteases. In addition, the inhibitory effect of ZnO NPs on the QS regulatory genes *lasI*, *lasR*, *rhlI*, *rhlR*, *pqsA*, and *pqsR*, which control the secretion of virulence factors, was confirmed (Saleh et al., 2019).

Further, *norA*, *norB*, *norC*, and *tet38* are important efflux pump genes that contribute to the MDR phenotype in *S. aureus*. The simultaneous use of ZnO@glutamic acid–thiosemicarbazide NP at sub-MIC concentrations in combination with ciprofloxacin decreased the expression of *norA*, *norB*, *norC*, and *tet38* by 5.4-, 3.8-, 2.1-, and 3.4-fold, respectively, compared to ciprofloxacin alone (Nejabatdoust et al., 2019).

A previous study showed that ZnO/zeolite NC at concentrations below the MIC in combination with chitosan reduced the expression of *gtfB*, *gtfC*, and *ftf* genes in *S. mutans* by 4.16-, 4.40-, and 2.88-fold, respectively (Afrasiabi et al., 2021). Along these lines, inhibition of the *esp* gene five-fold in *Enterococcus faecalis* decreased the ability to form biofilm (Partoazar et al., 2019). According to Khan et al. (2016), the expression level of the *gtfB* gene decreased 0.93-fold when *Streptococcus mitis* was grown with 30 μg/mL ZnO NP. Abdelghafar et al. also found a significant decrease in *icaA* and *sarA* genes after treatment of *S. aureus* with ZnO NPs (Abdelghafar et al., 2022). Similarly, sub-MIC of ZnO NPs had a significant effect on the biofilm formation rate of *S. aureus* and the gene expression of *ica A*, *ica D*, and *fnb A* (Abdelraheem et al., 2021).

### 7.3 TiO_2_ NPs

TiO_2_ NPs have been found as antimicrobial agents against various microorganisms that exert anti-QS activity. TiO_2_ NPs exhibited improved antibacterial and anti-biofilm activity in comparison to bulk form. In addition, it reduces expression of the efflux pump genes (*MexY*, *MexB*, *MexA*) and QS-regulated genes (*lasR*, *lasI*, *rhll*, *rhlR*, *pqsA*, *pqsR*) of *P. aeruginosa*. TiO_2_ NPs can increase the effectiveness of conventional antibiotics (Ahmed et al., 2021). The diminutive size, large surface area, and their ability to penetrate the cell wall are the factors determining the antimicrobial activities (Saranya et al., 2018). Moreover, exposure to UV light leads to the generation of ROS, which may enhance the antibacterial activity of TiO_2_ NPs (de Dicastillo et al., 2019). Several studies reported the antibacterial and antifungal activities of TiO_2_ NPs (Anupong et al., 2023; Younis et al., 2023). Abdulazeem et al. found that biofilm growth is completely reduced in *A. baumannii*, *P. aeruginosa*, *Proteus vulgaris*, and *Serratia marcescens* as TiO_2_ NPs target sulfhydryl groups in the cell membrane to form S–TiO_2_ bond. This reaction inhibits the electron transport chain and enzymes needed for biofilm formation (Abdulazeem et al., 2019). However, Abdel-Fatah et al. stated that TiO_2_ NPs had no bactericidal effect against *S. aureus*, *E. coli*, and *Bacillus subtilis* (Abdel-Fatah et al., 2016). These variations among different results may reflect differences in concentration, zeta potential, particle shape, size, and the examined pathogen (Skandalis et al., 2017).

The overexpression of efflux pump genes was dramatic in non-treated *P. aeruginosa*, especially the *MexY* gene. TiO_2_ NPs significantly reduce the expression of efflux pump genes with its efflux pump inhibitor activity (Ahmed et al., 2021). The *mxdABCD* complex is a new set of genes that are important essential for biofilm formation and growth and can be influenced by TiO_2_ NPs. Of these, *mxdA* gene encoding diguanylate cyclase and *mxdB* gene encoding glycosyl transferase, both of which confer to cell attachment and EPS synthesis. These genes are required for the three-dimensional of biofilm architecture (Maurer-Jones et al., 2013). In Liu et al. (2015) reported that TiO_2_ NPs on the surface and inside titania nanotubes reduced *gtfB*, *gtfC*, and *gtfD* genes of *S. mutans*, which improves the efficacy of orthopedic and dental implants. In a recent research, the antibacterial activity of TiO_2_ NPs and *Ganoderma* extract against *P. aeruginosa* and methicillin-resistant *Staphylococcus aureus* (MRSA) was investigated. As a result, the *algD* gene was reduced when TiO_2_ NPs were used alone or in combination with *Ganoderma* extract. None of them affected the *iacA* gene (Marzhoseyni et al., 2023). However, DNA damage induced by TiO_2_ NPs limits the efficacy of them (Chen et al., 2014).

### 7.4 Cu NPs

Cu NPs have proven to be efficient antibacterial agents due to their low cost, ease of mixing with polarized liquids such as water, and their relatively stable physical and chemical properties (LewisOscar et al., 2015). Graphene oxide-Cu NC has been shown to alter biofilm architecture, inhibit EPS formation and distribution, and dysregulate the expression of EPS-related genes (Mao et al., 2021). The *gtf* gene family, a glucosyltransferase (Gtf) encoding gene in *S. mutans*, can synthesize EPSs directly from sucrose. GTF-produced glucan maintains the integrity of the matrix structure. In exposure to graphene oxide-Cu NCs, the expression of *gtfB*, *gtfC*, and *gbpB* decreased, while the expression of the *rnc* gene increased. In addition, the expression of *vicRKX* was suppressed by *rnc* genes. Cell wall homeostasis and expression of *gtfB/C* genes can be enhanced by the VicRK two-component system. The group treated with graphene oxide and Cu NCs showed a significant increase in *rnc* expression, which is consistent with the negative effect of the *rnc* gene (Mao et al., 2021). Further, it was also reported that Cu/graphitic carbon nitride NCs downregulated *icaA* gene of *S. aureus* to reduce biofilm formation (Rashki et al., 2023b).

Cu NPs lowered the biofilm of *P. aeruginosa* by reducing the *ppyR* gene. In addition to this, the presence of Cu NPs led to an increase in the expression of the biofilm dispersion locus gene (*bdlA*). Also, Cu NPs caused a decrease in the *rsaL* gene as well as the *mexA* and *mex B* efflux genes (Singh et al., 2019). Aziz et al. found that the virulence gene-related to adhesion (*fimH*, *papC*) of *Klebsiella oxytoca* was reduced 8.5- and 9.0-fold by using Cu cobalt oxide NPs (Aziz et al., 2023).

The high toxicity of Cu NPs causes oxidative lesions, which limits the effectiveness of these NPs (Naz et al., 2020). Quercetin is a natural substance with anti-QS potential that has anti-biofilm properties. However, it has some disadvantages, including low water solubility and bioavailability issues. Quercetin/Cu NPs have been found to prevent biofilm formation by disrupting the integrity of the cell membrane and processes involved in QS, and by reducing the expression of *lasI*, *lasR*, *rhlI*, and *rhIR* genes in *P. aeruginosa* and *agrA* and *icaA* in *S. aureus*, and are non-toxic to mouse fibroblast cells (Cheng et al., 2024).

### 7.5 Fe_3_O_4_ NPs

Fe_3_O_4_ NPs have attracted attention due to their unique properties, which include larger surface area, superparamagnetic nature, surface-to-volume ratio, and easy separation process. These NPs have antibacterial and anti-biofilm properties via various mechanisms such as intracellular ROS generation, electrostatic attraction with the cell membrane and its proteins, leading to physical destruction and eventual death of the microbes. In addition, there are a number of advantages, including low toxicity, biocompatibility for medical applications and environmental friendliness (Das et al., 2021).

The clinical management of antibiotic-resistant *S. aureus* and *E. coli* raises a number of public health issues. Furthermore, increasingly serious antimicrobial resistance and biofilm formation pose a barrier to conventional therapy. Rhamnolipid (RHL)-coated Fe_3_O_4_ NPs- *p*-coumaric acid (*p*-CoA) and gallic acid (GA)-polymer common coatings (PVA; RHL-Fe_3_O_4_@PVA@*p*-CoA/GA) can strangely inhibit bacterial growth and biofilm formation via downregulating *icaABCD* and *csgBAC* operons, which encode the production of slime layer (*icaA* and *icaD*) and the production of curli fimbriae (*csgA*, *csgD*, and *crl*) in *S. aureus* and *E. coli*, respectively (Sharaf et al., 2022). Analysis of *fimA* and *csgA* genes for biofilm formation of *E. coli* confirmed the ability of clay halloysite nanotubules with tannic acid and Fe_3_O_4_ NPs to prevent bacterial adhesion and biofilm formation (Bu et al., 2024). A summary of the effect of the other NPs on the downregulation of bacterial biofilm-related genes can be found in Table 2.

**Table 2:** Summary of the effect of the other nanoparticles on the downregulation of bacterial biofilm-related genes.

Compound name	Target microorganisms	Target genes	Applications	References
Au NP + CEs	*Streptococcus agalactiae*	*agrA*	QSI	Fernando et al. (2020)
Au NP and Se NP	*Pseudomonas aeruginosa*	*lasI*, *lasR*, *rhlI*, *rhlR*, *pqsA*, and *pqsR*	QSI	Elshaer and Shaaban (2021)
CS NP	*P. aeruginosa*	*LasI*, *RhlI*	QSI	Fattah et al. (2021)
CS NP/LL37	MRSA	*icaA*	Anti-biofilm effect	Rashki et al. (2022)
CS/ZnO/LL37-NP	MRSA	*icaA*	Anti-biofilm effect	Rashki et al. (2023a)
CS-propolis NP	*Enterococcus faecalis*	*gelE*, *ace*, *asa*, *fsrB, fsrC*, *ebpA*, *ebpB*, *ebpC*, *efa*, *bopD*	Anti-biofilm effect	Ong et al. (2017)
CS-propolis NP	*S. epidermidis*	*rsbU*, *sarA*, *icaA,icaD,icaB,icaC*, *embp*, *atlE*	Anti-adhesion, Anti-biofilm effect	Ong et al. (2019)
MPEO- Chitosan NP	*Streptococcus mutans*	*gtfB, gtfC*, *gbpB*, *spaP*, *brpA*, *relA*, *vicR*	Anti-biofilm effect	Ashrafi et al. (2019)
Cur-Ag/Cu NP-Nio	*Staphylococcus aureus*-, *P. aeruginosa*	*ebp* - *arr*	Anti-biofilm effect	Targhi et al. (2021)
Nio -loaded Se NP	*S. aureus*-, *E. faecalis*-, *P. aeruginosa*-, *Escherichia coli*	*icaD*-, *ace*-, *fimH*-, *pelF*	Anti-biofilm effect	Haddadian et al. (2022)
SiO_2_ NP-lectin	*P. aeruginosa*	*rhlR*	Anti-biofilm effect	Nsayef Muslim et al. (2021)
Curcumin NP	*P. aeruginosa*	*algD*	Anti-biofilm effect	Rahimi et al. (2023)
LA/TCS@PLGA-NP	*S. mutans*	*gtfB, gtfC*, *ftf, gbpB, spaP*	Anti-biofilm effect	Weng et al. (2022)
DMSNs-NH_2_	*S. mutans*	*vicR*	EPS inhibition- Anti-biofilm effect	Tian et al. (2022)
Calcium fluoride NP	*S. mutans*	*vicR*, *gtfC*, *ftf*, *spaP*, *comDE*	Anti-biofilm effect	Kulshrestha et al. (2016)
GO-PEI-AS*yycG*	*S. aureus*	*icaA, icaD, icaB, icaC*	Anti-biofilm effect	Wu et al. (2019)
Polystyrene NP	*P. aeruginosa*	*pelA*, *rpsL*, *lasR*	EPS inhibition - QSI	Rodríguez-Suárez et al. (2023)
Selenium NP	*klebsiella pneumoniae*	*mrkA*	Anti-biofilm effect	Maleki et al. (2023)
Au NPs coated *Anthemis atropatana* extract	MDR-*K. pneumoniae*	*mrkA*, *wzm* *acrB*	Anti-biofilm effect- Anti-efflux pump	Khosravi et al. (2020)
BR-MPS NP	VRSA	*agr*, *clfA*, *pvl*, *tst*, *hla*, *icaA*	Anti-biofilm effect	Abd El-Hamid et al. (2024)
Cerium oxide NP	*P. aeruginosa*	*rhlI-rhlR*, *rhlAB*, *pqsR*-*pqsA*	QSI	Xu et al. (2018)
Samarium oxide NP	*P. aeruginosa*-*S. aureus*	*mexA*-, *mexB*, *norA*, *norB*	Anti-efflux pump	Zahmatkesh et al. (2023)

Au NP + CEs, ethnobotanical crude extracts + biologically synthesized gold nanoparticles; QSI, quorum sensing inhibition; Se, selenium; CS, chitosan; LL37, cathelicidin-derived antimicrobial peptide; ZnO, zinc oxide; MRSA, methicillin-resistant *Staphylococcus aureus*; MPEO, *Mentha piperita* essential oils; Cur-Ag/Cu NP-Nio, curcumin and silver/copper NP loaded noisome; Nio-loaded Se, noisome-loaded selenium; SiO_2_ NP-lectin, silica oxide NP-conjugation of lectin; LA/TCS@PLGA, triclosan-loaded poly (lactic-co-glycolic acid) with an envelope of *Lactobacillus*; DMSNs, aminated dendritic mesoporous silica nanoparticles; GO-PEI-AS*yycG*, nano graphene oxide-polyethyleneimine-green fluorescent protein; BR-MPS, berberine loaded mesoporous silica; VRSA, vancomycin-resistant *Staphylococcus aureus*.

Although metal NPs have been shown to be toxic in human cells, efforts are being focused on reducing their toxicity through various strategies, e.g., by producing composites with clays, doping with polymers and proteins, etc. (Odatsu et al., 2020). The limited use of NPs in clinical applications is due to the incomplete understanding of their side effects, and further studies are needed to utilize them more effectively. The routine use of NPs in the fight against bacterial infections will be possible once the toxicity of NPs is clarified by detailed *in vivo* and clinical studies. The correct determination of the threshold dose and exposure time is essential for the safe administration of NPs (Ozdal and Gurkok, 2022).

## 8 Conclusion

Reviewing the studies revealed that NPs target the gene expression and biomolecular of bacterial biofilm, resulting in destroying or hindering biofilm formation. NPs based on Ag NPs, ZnO NPs, TiO_2_ NPs, and Cu NPs are highly effective against biofilms of important pathogens due to their ability to inhibit *lasI*, *lasR*, *rhlI*, *rhlR* of *P. aeruginosa*, *gtfB*, *gtfC* of *S. mutans, and icaA* of *S. aureus*. Fe_3_O_4_ NPs reduced biofilm formation of *S. aureus* by inhibiting *icaA/D* expression. The versatility of NPs in combat with bacterial biofilm causes cells not to have enough opportunity to bypass or trigger the resistance mechanisms that are affected under gene regulation. For instance, NPs directly or via their derivatives like ROS or released ions, inhibit genes of virulence factors in bacteria. This phenomenon hinders the main factors that are necessary for the development and persistency of the biofilm. The bacterial genes associated with the flagella synthesis and other adhesion factors, penetration or internalization of vital elements, QS regulation, efflux pump, oxidative stress, etc. can be suppressed during exposure with the kind of NPs. Even though the antibacterial activity of NPs has been extensively studied, the mechanisms of action of them on biofilm regulatory genes are still poorly understood and controversial. So far, investigation of the mechanisms of action of NPs on the biofilm cells has developed targeted therapy and increased antibacterial efficacy. On the other hand, potential mutagenicity of the NPs may produce new dangerous antibiotic-resistant strains. The evolution of bacteria can be accelerated by NPs especially under co-culture in biofilm conditions. The use of NPs on an industrial scale can also have serious consequences for the environment which address specific issues to advance in research. As there is a necessity to develop new approaches to combat bacterial biofilms, new information about the efficacy of NPs against biofilms, the mechanisms of action on cells, mutagenicity, and genotoxicity will be also crucial.

## Author contributions

SA: Conceptualization, Writing – original draft, Writing – review & editing. AP: Conceptualization, Writing – review & editing.

## References

[ref1] Abd El-Hamid M. I. Ibrahim D. Elazab S. T. Gad W. M. Shalaby M. El-Neshwy W. M.. (2024). Tackling strong biofilm and multi-virulent vancomycin-resistant *Staphylococcus aureus* via natural alkaloid-based porous nanoparticles: perspective towards near future eradication. Front. Cell. Infect. Microbiol. 13:1287426. doi: 10.3389/fcimb.2023.1287426, PMID: 38282617 PMC10811083

[ref2] Abdel-Fatah W. I. Gobara M. M. Mustafa S. F. Ali G. W. Guirguis O. W. (2016). Role of silver nanoparticles in imparting antimicrobial activity of titanium dioxide. Mater. Lett. 179, 190–193. doi: 10.1016/j.matlet.2016.05.063

[ref3] Abdelghafar A. Yousef N. Askoura M. (2022). Zinc oxide nanoparticles reduce biofilm formation, synergize antibiotics action and attenuate *Staphylococcus aureus* virulence in host; an important message to clinicians. BMC Microbiol. 22:244. doi: 10.1186/s12866-022-02658-z, PMID: 36221053 PMC9552502

[ref4] Abdelraheem W. M. Abdelkader A. E. Mohamed E. S. Mohammed M. S. (2020). Detection of biofilm formation and assessment of biofilm genes expression in different *Pseudomonas aeruginosa* clinical isolates. Meta Gene 23:100646. doi: 10.1016/j.mgene.2020.100646

[ref5] Abdelraheem W. M. Khairy R. M. Zaki A. I. Zaki S. H. (2021). Effect of ZnO nanoparticles on methicillin, vancomycin, linezolid resistance and biofilm formation in *Staphylococcus aureus* isolates. Ann. Clin. Microbiol. Antimicrob. 20:54. doi: 10.1186/s12941-021-00459-2, PMID: 34419054 PMC8379777

[ref6] Abdulazeem L. Al-Amiedi B. Alrubaei H. A. Al-Mawlah Y. H. (2019). Titanium dioxide nanoparticles as antibacterial agents against some pathogenic bacteria. Drug Invent. Today 12, 963–967.

[ref7] Afrasiabi S. Bahador A. Partoazar A. (2021). Combinatorial therapy of chitosan hydrogel-based zinc oxide nanocomposite attenuates the virulence of *Streptococcus mutans*. BMC Microbiol. 21:62. doi: 10.1186/s12866-021-02128-y 33622240 PMC7903727

[ref8] Ahmed F. Y. Aly U. F. Abd El-Baky R. M. Waly N. G. (2021). Effect of titanium dioxide nanoparticles on the expression of efflux pump and quorum-sensing genes in Mdr *Pseudomonas aeruginosa* isolates. Antibiotics 10:625. doi: 10.3390/antibiotics10060625, PMID: 34073802 PMC8225175

[ref9] Alav I. Sutton J. M. Rahman K. M. (2018). Role of bacterial efflux pumps in biofilm formation. J. Antimicrob. Chemother. 73, 2003–2020. doi: 10.1093/jac/dky042 29506149

[ref10] Alshammari M. Ahmad A. Alkhulaifi M. Al Farraj D. Alsudir S. Alarawi M.. (2023). Reduction of biofilm formation of *Escherichia coli* by targeting quorum sensing and adhesion genes using the Crispr/Cas9-Hdr approach, and its clinical application on urinary catheter. J. Infect. Public Health 16, 1174–1183. doi: 10.1016/j.jiph.2023.05.026, PMID: 37271098

[ref11] Anupong W. On-Uma R. Jutamas K. Salmen S. H. Alharbi S. A. Joshi D.. (2023). Antibacterial, antifungal, antidiabetic, and antioxidant activities potential of Coleus aromaticus synthesized titanium dioxide nanoparticles. Environ. Res. 216:114714. doi: 10.1016/j.envres.2022.114714, PMID: 36334834

[ref12] Arora A. (2024). Crispri-mediated gene silencing in biofilm cycle and quorum sensing. Gene Editing in Plants: Crispr-Cas and Its Applications, 139–178. doi: 10.1007/978-981-99-8529-6_6

[ref13] Ashrafi B. Rashidipour M. Marzban A. Soroush S. Azadpour M. Delfani S.. (2019). Mentha piperita essential oils loaded in a chitosan nanogel with inhibitory effect on biofilm formation against *S. mutans* on the dental surface. Carbohydr. Polym. 212, 142–149. doi: 10.1016/j.carbpol.2019.02.018, PMID: 30832841

[ref14] Askoura M. Almalki A. J. Lila A. S. A. Almansour K. Alshammari F. Khafagy E.-S.. (2021). Alteration of *Salmonella enterica* virulence and host pathogenesis through targeting sdiA by using the Crispr-Cas9 system. Microorganisms 9:2564. doi: 10.3390/microorganisms9122564, PMID: 34946165 PMC8707642

[ref15] Asma S. T. Imre K. Morar A. Herman V. Acaroz U. Mukhtar H.. (2022). An overview of biofilm formation–combating strategies and mechanisms of action of antibiofilm agents. Lifestyles 12:1110. doi: 10.3390/life12081110, PMID: 35892912 PMC9394423

[ref16] Aziz S. N. Al-Kadmy I. M. Rheima A. M. Al-Sallami K. J. Abd Ellah N. H. El-Saber Batiha G.. (2023). Binary CuO\CoO nanoparticles inhibit biofilm formation and reduce the expression of papC and fimH genes in multidrug-resistant *Klebsiella oxytoca*. Mol. Biol. Rep. 50, 5969–5976. doi: 10.1007/s11033-023-08447-9, PMID: 37269387

[ref17] Baig N. Kammakakam I. Falath W. (2021). Nanomaterials: a review of synthesis methods, properties, recent progress, and challenges. Mater. Adv 2, 1821–1871. doi: 10.1039/D0MA00807A

[ref18] Bhargava A. Pareek V. Roy Choudhury S. Panwar J. Karmakar S. (2018). Superior bactericidal efficacy of fucose-functionalized silver nanoparticles against *Pseudomonas aeruginosa* Pao1 and prevention of its colonization on urinary catheters. Acs Appl Bio Mater 10, 29325–29337. doi: 10.1021/acsami.8b09475, PMID: 30096228

[ref19] Bu K.-B. Kim M. Sung J.-S. Kadam A. A. (2024). Halloysite nanotubes decorated with Fe3O4 nanoparticles and tannic acid for effective inhibition of *E. coli* biofilm. Acs Appl Nano Mater 7, 1, 313–322. doi: 10.1021/acsanm.3c04518

[ref20] Campoccia D. Montanaro L. Arciola C. R. (2013). A review of the biomaterials technologies for infection-resistant surfaces. Biomaterials 34, 8533–8554. doi: 10.1016/j.biomaterials.2013.07.089, PMID: 23953781

[ref21] Cangui-Panchi S. P. Ñacato-Toapanta A. L. Enríquez-Martínez L. J. Reyes J. Garzon-Chavez D. Machado A. (2022). Biofilm-forming microorganisms causing hospital-acquired infections from intravenous catheter: a systematic review. Curr Res Microb Sci. 3:100175. doi: 10.1016/j.crmicr.2022.100175 PMC9743049 36518176

[ref22] Chakraborty P. Paul P. Kumari M. Bhattacharjee S. Singh M. Maiti D.. (2021). Attenuation of *Pseudomonas aeruginosa* biofilm by thymoquinone: an individual and combinatorial study with tetrazine-capped silver nanoparticles and tryptophan. Folia Microbiol. 66, 255–271. doi: 10.1007/s12223-020-00841-1, PMID: 33411249

[ref23] Chen Z. Wang Y. Ba T. Li Y. Pu J. Chen T.. (2014). Genotoxic evaluation of titanium dioxide nanoparticles in vivo and in vitro. Toxicol. Lett. 226, 314–319. doi: 10.1016/j.toxlet.2014.02.020, PMID: 24594277

[ref24] Cheng J. Zhang H. Lu K. Zou Y. Jia D. Yang H.. (2024). Bi-functional quercetin/copper nanoparticles integrating bactericidal and anti-quorum sensing properties for preventing the formation of biofilms. Biomater. Sci. 12, 1788–1800. doi: 10.1039/D4BM00034J, PMID: 38390988

[ref25] Colino C. I. Millán C. G. Lanao J. M. (2018). Nanoparticles for signaling in biodiagnosis and treatment of infectious diseases. Int. J. Mol. Sci. 19:1627. doi: 10.3390/ijms19061627, PMID: 29857492 PMC6032068

[ref26] Czaczyk K. Myszka K. (2007). Biosynthesis of extracellular polymeric substances (eps) and its role in microbial biofilm formation. Pol. J. Environ. Stud. 16, 799–806.

[ref27] Das P. Ghosh S. Nayak B. (2021). Phyto-fabricated nanoparticles and their anti-biofilm activity: Progress and current status. Front Nanotechnol 3:739286. doi: 10.3389/fnano.2021.739286

[ref28] De Dicastillo C. L. Patiño C. Galotto M. J. Vásquez-Martínez Y. Torrent C. Alburquenque D.. (2019). Novel hollow titanium dioxide nanospheres with antimicrobial activity against resistant bacteria. Beilstein J Nanotechnol 10, 1716–1725. doi: 10.3762/bjnano.10.167, PMID: 31501743 PMC6720579

[ref29] De Romana D. L. Brown K. Guinard J. X. (2002). Sensory trial to assess the acceptability of zinc fortificants added to iron-fortified wheat products. J. Food Sci. 67, 461–465. doi: 10.1111/j.1365-2621.2002.tb11429.x

[ref30] Dolatabadi A. Noorbazargan H. Khayam N. Moulavi P. Zamani N. Asghari Lalami Z.. (2021). Ecofriendly biomolecule-capped *Bifidobacterium bifidum*-manufactured silver nanoparticles and efflux pump genes expression alteration in *Klebsiella pneumoniae*. Microb. Drug Resist. 27, 247–257. doi: 10.1089/mdr.2019.0366, PMID: 32635796

[ref31] Elshaer S. L. Shaaban M. I. (2021). Inhibition of quorum sensing and virulence factors of *Pseudomonas aeruginosa* by biologically synthesized gold and selenium nanoparticles. Antibiotics 10:1461. doi: 10.3390/antibiotics10121461, PMID: 34943673 PMC8698379

[ref32] Elwakil B. H. Eldrieny A. M. Almotairy A. R. Z. El-Khatib M. (2024). Potent biological activity of newly fabricated silver nanoparticles coated by a carbon shell synthesized by electrical arc. Sci. Rep. 14, 1–12. doi: 10.1038/s41598-024-54648-y 38438447 PMC10912099

[ref33] Fattah R. A. F. A. Mohamed T. A. H. Elsayed M. S. (2021). Effect of chitosan nanoparticles on quorum sensing-controlled virulence factors and expression of LasI and RhlI genes among *Pseudomonas aeruginosa* clinical isolates. Aims microbiol 7, 415–430. doi: 10.3934/microbiol.2021025, PMID: 35071940 PMC8712529

[ref34] Fernando S. I. D. Judan Cruz K. G. Watanabe K. (2020). Quorum sensing-linked agrA expression by ethno-synthesized gold nanoparticles in Tilapia *Streptococcus agalactiae* biofilm formation. BioNanoScience 10, 696–704. doi: 10.1007/s12668-020-00758-6

[ref35] Flemming H.-C. (2016). Eps—then and now. Microorganisms 4:41. doi: 10.3390/microorganisms4040041, PMID: 27869702 PMC5192524

[ref36] Foroohimanjili F. Mirzaie A. Hamdi S. M. M. Noorbazargan H. Hedayati Ch M. Dolatabadi A.. (2020). Antibacterial, antibiofilm, and antiquorum sensing activities of phytosynthesized silver nanoparticles fabricated from *Mespilus germanica* extract against multidrug resistance of *Klebsiella pneumoniae* clinical strains. J. Basic Microbiol. 60, 216–230. doi: 10.1002/jobm.201900511, PMID: 31994223

[ref37] Gestel J. V. Vlamakis H. Kolter R. (2015). Division of labor in biofilms: the ecology of cell differentiation. Microbiol Spectr 3:MB-0002-2014. doi: 10.1128/microbiolspec.MB-0002-2014 26104716

[ref38] Gupta P. Sarkar S. Das B. Bhattacharjee S. Tribedi P. (2016). Biofilm, pathogenesis and prevention—a journey to break the wall: a review. Arch. Microbiol. 198, 1–15. doi: 10.1007/s00203-015-1148-6, PMID: 26377585

[ref39] Gupta D. Singh A. Khan A. U. (2017). Nanoparticles as efflux pump and biofilm inhibitor to rejuvenate bactericidal effect of conventional antibiotics. Nanoscale Res. Lett. 12:454. doi: 10.1186/s11671-017-2222-6 28709374 PMC5509568

[ref40] Haddadian A. Robattorki F. F. Dibah H. Soheili A. Ghanbarzadeh E. Sartipnia N.. (2022). Niosomes-loaded selenium nanoparticles as a new approach for enhanced antibacterial, anti-biofilm, and anticancer activities. Sci. Rep. 12:21938. doi: 10.1038/s41598-022-26400-x, PMID: 36536030 PMC9763330

[ref41] Hamida R. S. Ali M. A. Goda D. A. Khalil M. I. Al-Zaban M. I. (2020). Novel biogenic silver nanoparticle-induced reactive oxygen species inhibit the biofilm formation and virulence activities of methicillin-resistant *Staphylococcus aureus* (Mrsa) strain. Front. Bioeng. Biotechnol. 8:433. doi: 10.3389/fbioe.2020.00433, PMID: 32548095 PMC7270459

[ref42] Hemeg H. A. (2017). Nanomaterials for alternative antibacterial therapy. Int. J. Nanomedicine 12, 8211–8225. doi: 10.2147/IJN.S132163, PMID: 29184409 PMC5689025

[ref43] Hetta H. F. Al-Kadmy I. M. Khazaal S. S. Abbas S. Suhail A. El-Mokhtar M. A.. (2021). Antibiofilm and antivirulence potential of silver nanoparticles against multidrug-resistant *Acinetobacter baumannii*. Sci. Rep. 11:10751. doi: 10.1038/s41598-021-90208-4, PMID: 34031472 PMC8144575

[ref44] Holling N. Lednor D. Tsang S. Bissell A. Campbell L. Nzakizwanayo J.. (2014). Elucidating the genetic basis of crystalline biofilm formation in *Proteus mirabilis*. Infect. Immun. 82, 1616–1626. doi: 10.1128/IAI.01652-13, PMID: 24470471 PMC3993403

[ref45] Honarmand T. Sharif A. P. Salehzadeh A. Jalali A. Nikokar I. (2022). Does conjugation of silver nanoparticles with thiosemicarbazide increase their antibacterial properties? Microb. Drug Resist. 28, 293–305. doi: 10.1089/mdr.2020.0557, PMID: 35005985

[ref46] Hong K.-W. Koh C.-L. Sam C.-K. Yin W.-F. Chan K.-G. (2012). Quorum quenching revisited—from signal decays to signalling confusion. Sensors 12, 4661–4696. doi: 10.3390/s120404661, PMID: 22666051 PMC3355433

[ref47] Hou J. Wu Y. Li X. Wei B. Li S. Wang X. (2018). Toxic effects of different types of zinc oxide nanoparticles on algae, plants, invertebrates, vertebrates and microorganisms. Chemosphere 193, 852–860. doi: 10.1016/j.chemosphere.2017.11.077, PMID: 29874759

[ref48] Huang Q. Wang S. Sun Y. Shi C. Yang H. Lu Z. (2020). Effects of ag/ZnO nanocomposite at sub-minimum inhibitory concentrations on virulence factors of *Streptococcus mutans*. Arch. Oral Biol. 111:104640. doi: 10.1016/j.archoralbio.2019.104640, PMID: 31884336

[ref49] Jena P. Mohanty S. Mallick R. Jacob B. Sonawane A. (2012). Toxicity and antibacterial assessment of chitosancoated silver nanoparticles on human pathogens and macrophage cells. Int. J. Nanomedicine 7, 1805–1818. doi: 10.2147/IJN.S28077, PMID: 22619529 PMC3356211

[ref50] Jiang Y. Geng M. Bai L. (2020). Targeting biofilms therapy: current research strategies and development hurdles. Microorganisms 8:1222. doi: 10.3390/microorganisms8081222, PMID: 32796745 PMC7465149

[ref51] Jin J. Zhang L. Shi M. Zhang Y. Wang Q. (2017). Ti-go-ag nanocomposite: the effect of content level on the antimicrobial activity and cytotoxicity. Int. J. Nanomedicine 12, 4209–4224. doi: 10.2147/IJN.S134843, PMID: 28652728 PMC5473600

[ref52] Kalishwaralal K. Barathmanikanth S. Pandian S. R. K. Deepak V. Gurunathan S. (2010). Silver nanoparticles impede the biofilm formation by Pseudomonas aeruginosa and *Staphylococcus epidermidis*. Colloids Surf. B: Biointerfaces 79, 340–344. doi: 10.1016/j.colsurfb.2010.04.014, PMID: 20493674

[ref53] Karatan E. Watnick P. (2009). Signals, regulatory networks, and materials that build and break bacterial biofilms. Microbiol. Mol. Biol. Rev. 73, 310–347. doi: 10.1128/MMBR.00041-08, PMID: 19487730 PMC2698413

[ref54] Khan S. T. Ahmad J. Ahamed M. Musarrat J. Al-Khedhairy A. A. (2016). Zinc oxide and titanium dioxide nanoparticles induce oxidative stress, inhibit growth, and attenuate biofilm formation activity of *Streptococcus mitis*. J. Biol. Inorg. Chem. 21, 295–303. doi: 10.1007/s00775-016-1339-x, PMID: 26837748

[ref55] Khosravi M. Mirzaie A. Kashtali A. B. Noorbazargan H. (2020). Antibacterial, anti-efflux, anti-biofilm, anti-slime (exopolysaccharide) production and urease inhibitory efficacies of novel synthesized gold nanoparticles coated Anthemis atropatana extract against multidrug-resistant *Klebsiella pneumoniae* strains. Arch. Microbiol. 202, 2105–2115. doi: 10.1007/s00203-020-01930-y, PMID: 32500253

[ref56] Kot B. Sytykiewicz H. Sprawka I. (2018). Expression of the biofilm-associated genes in methicillin-resistant *Staphylococcus aureus* in biofilm and planktonic conditions. Int. J. Mol. Sci. 19:3487. doi: 10.3390/ijms19113487, PMID: 30404183 PMC6274806

[ref57] Kulshrestha S. Khan S. Hasan S. Khan M. E. Misba L. Khan A. U. (2016). Calcium fluoride nanoparticles induced suppression of *Streptococcus mutans* biofilm: an in vitro and in vivo approach. Appl. Microbiol. Biotechnol. 100, 1901–1914. doi: 10.1007/s00253-015-7154-4, PMID: 26610805

[ref58] Kumar S. Paliya B. S. Singh B. N. (2022). Superior inhibition of virulence and biofilm formation of *Pseudomonas aeruginosa* Pao1 by phyto-synthesized silver nanoparticles through anti-quorum sensing activity. Microb. Pathog. 170:105678. doi: 10.1016/j.micpath.2022.105678, PMID: 35820580

[ref59] Lahiri D. Nag M. Sheikh H. I. Sarkar T. Edinur H. A. Pati S.. (2021). Microbiologically-synthesized nanoparticles and their role in silencing the biofilm signaling cascade. Front. Microbiol. 12:636588. doi: 10.3389/fmicb.2021.636588, PMID: 33717030 PMC7947885

[ref60] Le Ouay B. Stellacci F. (2015). Antibacterial activity of silver nanoparticles: a surface science insight. Nano Today 10, 339–354. doi: 10.1016/j.nantod.2015.04.002

[ref61] Lee S. H. Jun B.-H. (2019). Silver nanoparticles: synthesis and application for nanomedicine. Int. J. Mol. Sci. 20:865. doi: 10.3390/ijms20040865, PMID: 30781560 PMC6412188

[ref62] Lewisoscar F. Mubarakali D. Nithya C. Priyanka R. Gopinath V. Alharbi N. S.. (2015). One pot synthesis and anti-biofilm potential of copper nanoparticles (Cunps) against clinical strains of *Pseudomonas aeruginosa*. Biofouling 31, 379–391. doi: 10.1080/08927014.2015.1048686, PMID: 26057498

[ref63] Li C.-H. Shen C.-C. Cheng Y.-W. Huang S.-H. Wu C.-C. Kao C.-C.. (2012). Organ biodistribution, clearance, and genotoxicity of orally administered zinc oxide nanoparticles in mice. Nanotoxicology 6, 746–756. doi: 10.3109/17435390.2011.620717, PMID: 21950449

[ref64] Li F. Weir M. D. Chen J. Xu H. H. (2013). Comparison of quaternary ammonium-containing with nano-silver-containing adhesive in antibacterial properties and cytotoxicity. Dent. Mater. 29, 450–461. doi: 10.1016/j.dental.2013.01.012, PMID: 23428077 PMC3631003

[ref65] Liao C. Li Y. Tjong S. C. (2019). Bactericidal and cytotoxic properties of silver nanoparticles. Int. J. Mol. Sci. 20:449. doi: 10.3390/ijms20020449, PMID: 30669621 PMC6359645

[ref66] Liu L. Li J.-H. Zi S.-F. Liu F.-R. Deng C. Ao X.. (2019). Agnp combined with quorum sensing inhibitor increased the antibiofilm effect on *Pseudomonas aeruginosa*. Appl. Microbiol. Biotechnol. 103, 6195–6204. doi: 10.1007/s00253-019-09905-w, PMID: 31129741

[ref67] Liu W. Su P. Chen S. Wang N. Wang J. Liu Y.. (2015). Antibacterial and osteogenic stem cell differentiation properties of photoinduced TiO2 nanoparticle-decorated TiO2 nanotubes. Nanomedicine 10, 713–723. doi: 10.2217/nnm.14.183, PMID: 25816875

[ref68] Mahamuni-Badiger P. P. Patil P. M. Badiger M. V. Patel P. R. Thorat-Gadgil B. S. Pandit A.. (2020). Biofilm formation to inhibition: role of zinc oxide-based nanoparticles. Mater. Sci. Eng. C Mater. Biol. Appl. 108:110319. doi: 10.1016/j.msec.2019.110319, PMID: 31923962

[ref69] Maleki A. R. Tabatabaei R. R. Aminian F. Ranjbar S. Ashrafi F. Ranjbar R. (2023). Antibacterial and antibiofilm effects of green synthesized selenium nanoparticles on clinical *Klebsiella pneumoniae* isolates. J. Basic Microbiol. 63, 1373–1382. doi: 10.1002/jobm.202300332, PMID: 37699755

[ref70] Mao M. Zhang W. Huang Z. Huang J. Wang J. Li W.. (2021). Graphene oxide-copper nanocomposites suppress cariogenic *Streptococcus mutans* biofilm formation. Int. J. Nanomedicine 16, 7727–7739. doi: 10.2147/IJN.S303521, PMID: 34824531 PMC8610231

[ref71] Marzhoseyni Z. Rashki S. Nazari-Alam A. (2023). Evaluation of the inhibitory effects of TiO2 nanoparticle and Ganoderma lucidum extract against biofilm-producing bacteria isolated from clinical samples. Arch. Microbiol. 205:59. doi: 10.1007/s00203-023-03403-4, PMID: 36622472

[ref72] Maurer-Jones M. A. Gunsolus I. L. Meyer B. M. Christenson C. J. Haynes C. L. (2013). Impact of TiO2 nanoparticles on growth, biofilm formation, and flavin secretion in *Shewanella oneidensis*. Anal. Chem. 85, 5810–5818. doi: 10.1021/ac400486u, PMID: 23701037 PMC3733218

[ref73] May T. Ito A. Okabe S. (2009). Induction of multidrug resistance mechanism in *Escherichia coli* biofilms by interplay between tetracycline and ampicillin resistance genes. Antimicrob. Agents Chemother. 53, 4628–4639. doi: 10.1128/AAC.00454-09, PMID: 19721076 PMC2772303

[ref74] Miola M. Fucale G. Maina G. Verne E. (2015). Antibacterial and bioactive composite bone cements containing surface silver-doped glass particles. Biomed. Mater. 10:055014. doi: 10.1088/1748-6041/10/5/055014, PMID: 26481324

[ref75] Mohanta Y. K. Chakrabartty I. Mishra A. K. Chopra H. Mahanta S. Avula S. K.. (2023). Nanotechnology in combating biofilm: a smart and promising therapeutic strategy. Front. Microbiol. 13:1028086. doi: 10.3389/fmicb.2022.1028086, PMID: 36938129 PMC10020670

[ref76] Montazeri A. Salehzadeh A. Zamani H. (2020). Effect of silver nanoparticles conjugated to thiosemicarbazide on biofilm formation and expression of intercellular adhesion molecule genes, icaad, in *Staphylococcus aureus*. Folia Microbiol. 65, 153–160. doi: 10.1007/s12223-019-00715-1, PMID: 31114932

[ref77] Moulavi P. Noorbazargan H. Dolatabadi A. Foroohimanjili F. Tavakoli Z. Mirzazadeh S.. (2019). Antibiofilm effect of green engineered silver nanoparticles fabricated from Artemisia scoporia extract on the expression of icaA and icaR genes against multidrug-resistant *Staphylococcus aureus*. J. Basic Microbiol. 59, 701–712. doi: 10.1002/jobm.201900096, PMID: 31032943

[ref78] Mousavi S. M. Mousavi S. M. Moeinizadeh M. Aghajanidelavar M. Rajabi S. Mirshekar M. (2023). Evaluation of biosynthesized silver nanoparticles effects on expression levels of virulence and biofilm-related genes of multidrug-resistant *Klebsiella pneumoniae* isolates. J. Basic Microbiol. 63, 632–645. doi: 10.1002/jobm.202200612, PMID: 36658772

[ref79] Muhammad M. H. Idris A. L. Fan X. Guo Y. Yu Y. Jin X.. (2020). Beyond risk: bacterial biofilms and their regulating approaches. Front. Microbiol. 11:928. doi: 10.3389/fmicb.2020.00928, PMID: 32508772 PMC7253578

[ref80] Naz S. Gul A. Zia M. (2020). Toxicity of copper oxide nanoparticles: a review study. IET Nanobiotechnol. 14, 1–13. doi: 10.1049/iet-nbt.2019.0176, PMID: 31935671 PMC8676634

[ref81] Nejabatdoust A. Zamani H. Salehzadeh A. (2019). Functionalization of ZnO nanoparticles by glutamic acid and conjugation with thiosemicarbazide alters expression of efflux pump genes in multiple drug-resistant *Staphylococcus aureus* strains. Microb. Drug Resist. 25, 966–974. doi: 10.1089/mdr.2018.0304, PMID: 30855211

[ref82] Nsayef Muslim S. Mohammed Ali A. N. Auda I. G. (2021). Anti-biofilm and anti-virulence effects of silica oxide nanoparticle–conjugation of lectin purified from *Pseudomonas aeruginosa*. IET Nanobiotechnol. 15, 318–328. doi: 10.1049/nbt2.12022, PMID: 34694672 PMC8675845

[ref83] Odatsu T. Kuroshima S. Sato M. Takase K. Valanezhad A. Naito M.. (2020). Antibacterial properties of nano-ag coating on healing abutment: an in vitro and clinical study. Antibiotics 9:347. doi: 10.3390/antibiotics9060347, PMID: 32575552 PMC7345643

[ref84] Ong T. H. Chitra E. Ramamurthy S. Ling C. C. S. Ambu S. P. Davamani F. (2019). Cationic chitosan-propolis nanoparticles alter the zeta potential of *S. epidermidis*, inhibit biofilm formation by modulating gene expression and exhibit synergism with antibiotics. PLoS One 14:e0213079. doi: 10.1371/journal.pone.0213079, PMID: 30818374 PMC6394969

[ref85] Ong T. H. Chitra E. Ramamurthy S. Siddalingam R. P. Yuen K. H. Ambu S. P.. (2017). Chitosan-propolis nanoparticle formulation demonstrates anti-bacterial activity against *Enterococcus faecalis* biofilms. PLoS One 12:e0174888. doi: 10.1371/journal.pone.0174888, PMID: 28362873 PMC5376299

[ref86] Ozdal M. Gurkok S. (2022). Recent advances in nanoparticles as antibacterial agent. Admet Dmpk 10, 115–129. doi: 10.5599/admet.1172, PMID: 35350114 PMC8957245

[ref87] Palmer J. Flint S. Brooks J. (2007). Bacterial cell attachment, the beginning of a biofilm. J. Ind. Microbiol. Biotechnol. 34, 577–588. doi: 10.1007/s10295-007-0234-4, PMID: 17619090

[ref88] Partoazar A. Talaei N. Bahador A. Pourhajibagher M. Dehpour S. Sadati M.. (2019). Antibiofilm activity of natural zeolite supported NanoZnO: inhibition of Esp gene expression of *Enterococcus faecalis*. Nanomedicine 14, 675–687. doi: 10.2217/nnm-2018-0173, PMID: 30702017

[ref89] Pourmbarak Mahnaie M. Mahmoudi H. (2020). Effect of glutathione-stabilized silver nanoparticles on expression of las I and las R of the genes in *Pseudomonas aeruginosa* strains. Eur. J. Med. Res. 25:17. doi: 10.1186/s40001-020-00415-4 32434568 PMC7238514

[ref90] Qayyum S. Khan A. U. (2016). Nanoparticles vs. biofilms: a battle against another paradigm of antibiotic resistance. Med. Chem. Commun. 7, 1479–1498. doi: 10.1039/C6MD00124F

[ref91] Rahimi M. Piroozmand A. Shayestehpour M. Salamat S. Peik Falak F. Shakerimoghaddam A.. (2023). Effect of curcumin nanoparticles and alcoholic extract of *Falcaria vulgaris* on the growth rate, biofilm, and gene expression in *Pseudomonas aeruginosa* isolated from burn wound infection. Mol. Biol. Rep. 50, 6681–6690. doi: 10.1007/s11033-023-08559-2, PMID: 37378742

[ref92] Rashki S. Dawi E. A. Zilaei M. R. Safardoust-Hojaghan H. Ghanbari M. Ryadh A.. (2023a). ZnO/chitosan nanocomposites as a new approach for delivery Ll37 and evaluation of the inhibitory effects against biofilm-producing methicillin-resistant *Staphylococcus aureus* isolated from clinical samples. Int. J. Biol. Macromol. 253:127583. doi: 10.1016/j.ijbiomac.2023.127583, PMID: 37866577

[ref93] Rashki S. Ghanbari M. Khudhair Z. H. Marzhoseyni Z. Bameri Z. Afsharikhah S.. (2023b). Evaluate the effect of graphitic carbon nitride nanosheets decorated with copper nanoparticles on biofilm and icaA gene expression in *Staphylococcus aureus* isolated from clinical samples. Arab. J. Chem. 16:104882. doi: 10.1016/j.arabjc.2023.104882

[ref94] Rashki S. Safardoust-Hojaghan H. Mirzaei H. Abdulsahib W. K. Mahdi M. A. Salavati-Niasari M.. (2022). Delivery Ll37 by chitosan nanoparticles for enhanced antibacterial and antibiofilm efficacy. Carbohydr. Polym. 291:119634. doi: 10.1016/j.carbpol.2022.119634, PMID: 35698353

[ref95] Ren D. Bedzyk L. Thomas S. Ye R. Wood T. K. (2004). Gene expression in *Escherichia coli* biofilms. Appl. Microbiol. Biotechnol. 64, 515–524. doi: 10.1007/s00253-003-1517-y 14727089

[ref96] Renner L. D. Weibel D. B. (2011). Physicochemical regulation of biofilm formation. MRS Bull. 36, 347–355. doi: 10.1557/mrs.2011.65, PMID: 22125358 PMC3224470

[ref97] Rodríguez-Suárez J. M. Gershenson A. Onuh T. U. Butler C. S. (2023). The heterogeneous diffusion of polystyrene nanoparticles and the effect on the expression of quorum-sensing genes and eps production as a function of particle charge and biofilm age. Environ. Sci. Nano 10, 2551–2565. doi: 10.1039/D3EN00219E, PMID: 37868332 PMC10585598

[ref98] Roy R. Tiwari M. Donelli G. Tiwari V. (2018). Strategies for combating bacterial biofilms: a focus on anti-biofilm agents and their mechanisms of action. Virulence 9, 522–554. doi: 10.1080/21505594.2017.1313372, PMID: 28362216 PMC5955472

[ref99] Sadekuzzaman M. Yang S. Mizan M. Ha S. (2015). Current and recent advanced strategies for combating biofilms. Compr. Rev. Food Sci. Food Saf. 14, 491–509. doi: 10.1111/1541-4337.12144

[ref100] Saleh M. M. Sadeq R.'. A. Abdel Latif H. K. Abbas H. A. Askoura M. (2019). Zinc oxide nanoparticles inhibits quorum sensing and virulence in *Pseudomonas aeruginosa*. Afr. Health Sci. 19, 2043–2055. doi: 10.4314/ahs.v19i2.28, PMID: 31656488 PMC6794539

[ref101] Sánchez P. Linares J. F. Ruiz-Díez B. Campanario E. Navas A. Baquero F.. (2002). Fitness of in vitro selected *Pseudomonas aeruginosa* nalB and nfxB multidrug resistant mutants. J. Antimicrob. Chemother. 50, 657–664. doi: 10.1093/jac/dkf185, PMID: 12407121

[ref102] Saranya K. S. Vellora Thekkae Padil V. Senan C. Pilankatta R. Saranya K. George B.. (2018). Green synthesis of high temperature stable anatase titanium dioxide nanoparticles using gum kondagogu: characterization and solar driven photocatalytic degradation of organic dye. Nano 8:1002. doi: 10.3390/nano8121002, PMID: 30518035 PMC6316888

[ref103] Shahverdi A. R. Fakhimi A. Shahverdi H. R. Minaian S. (2007). Synthesis and effect of silver nanoparticles on the antibacterial activity of different antibiotics against Staphylococcus aureus and *Escherichia coli*. Nanomedicine 3, 168–171. doi: 10.1016/j.nano.2007.02.001 17468052

[ref104] Sharaf M. Sewid A. H. Hamouda H. Elharrif M. G. El-Demerdash A. S. Alharthi A.. (2022). Rhamnolipid-coated iron oxide nanoparticles as a novel multitarget candidate against major foodborne *E. coli* serotypes and methicillin-resistant *S. aureus*. Microbiol Spectr 10, e00250–e00222. doi: 10.1128/spectrum.00250-22 35852338 PMC9430161

[ref105] Sharma D. Misba L. Khan A. U. (2019). Antibiotics versus biofilm: an emerging battleground in microbial communities. Antimicrob. Resist. Infect. Control 8:76. doi: 10.1186/s13756-019-0533-3 31131107 PMC6524306

[ref106] Shkodenko L. Kassirov I. Koshel E. (2020). Metal oxide nanoparticles against bacterial biofilms: perspectives and limitations. Microorganisms 8:1545. doi: 10.3390/microorganisms8101545, PMID: 33036373 PMC7601517

[ref107] Singh N. Paknikar K. M. Rajwade J. (2019). Gene expression is influenced due to ‘nano’and ‘ionic’copper in pre-formed *Pseudomonas aeruginosa* biofilms. Environ. Res. 175, 367–375. doi: 10.1016/j.envres.2019.05.034, PMID: 31153105

[ref108] Singh B. R. Singh B. N. Singh A. Khan W. Naqvi A. H. Singh H. B. (2015). Mycofabricated biosilver nanoparticles interrupt *Pseudomonas aeruginosa* quorum sensing systems. Sci. Rep. 5:13719. doi: 10.1038/srep13719, PMID: 26347993 PMC4562228

[ref109] Skandalis N. Dimopoulou A. Georgopoulou A. Gallios N. Papadopoulos D. Tsipas D.. (2017). The effect of silver nanoparticles size, produced using plant extract from *Arbutus unedo*, on their antibacterial efficacy. Nano 7:178. doi: 10.3390/nano7070178, PMID: 28698511 PMC5535244

[ref110] Sondi I. Salopek-Sondi B. (2004). Silver nanoparticles as antimicrobial agent: a case study on *E. coli* as a model for gram-negative bacteria. J. Colloid Interface Sci. 275, 177–182. doi: 10.1016/j.jcis.2004.02.012, PMID: 15158396

[ref111] Srinivasan R. Vigneshwari L. Rajavel T. Durgadevi R. Kannappan A. Balamurugan K.. (2018). Biogenic synthesis of silver nanoparticles using *Piper betle* aqueous extract and evaluation of its anti-quorum sensing and antibiofilm potential against uropathogens with cytotoxic effects: an in vitro and in vivo approach. Environ. Sci. Pollut. Res. Int. 25, 10538–10554. doi: 10.1007/s11356-017-1049-0, PMID: 29288300

[ref112] Swolana D. Kępa M. Kruszniewska-Rajs C. Wojtyczka R. D. (2022). Antibiofilm effect of silver nanoparticles in changing the biofilm-related gene expression of *Staphylococcus epidermidis*. Int. J. Mol. Sci. 23:9257. doi: 10.3390/ijms23169257, PMID: 36012520 PMC9409202

[ref113] Targhi A. A. Moammeri A. Jamshidifar E. Abbaspour K. Sadeghi S. Lamakani L.. (2021). Synergistic effect of curcumin-cu and curcumin-ag nanoparticle loaded niosome: enhanced antibacterial and anti-biofilm activities. Bioorg. Chem. 115:105116. doi: 10.1016/j.bioorg.2021.105116, PMID: 34333420

[ref114] Thuptimdang P. Limpiyakorn T. Mcevoy J. Pruß B. M. Khan E. (2015). Effect of silver nanoparticles on *Pseudomonas putida* biofilms at different stages of maturity. J. Hazard. Mater. 290, 127–133. doi: 10.1016/j.jhazmat.2015.02.073, PMID: 25756827

[ref115] Tian Y. Zhang Y. Zhang M. Chen X. Lei L. Hu T. (2022). Antisense vicR-loaded dendritic mesoporous silica nanoparticles regulate the biofilm organization and cariogenicity of *Streptococcus mutans*. Int. J. Nanomedicine 17, 1255–1272. doi: 10.2147/IJN.S334785, PMID: 35340824 PMC8956320

[ref116] Toyofuku M. Inaba T. Kiyokawa T. Obana N. Yawata Y. Nomura N. (2016). Environmental factors that shape biofilm formation. Biosci. Biotechnol. Biochem. 80, 7–12. doi: 10.1080/09168451.2015.1058701 26103134

[ref117] Uddin T. M. Chakraborty A. J. Khusro A. Zidan B. R. M. Mitra S. Emran T. B.. (2021). Antibiotic resistance in microbes: history, mechanisms, therapeutic strategies and future prospects. J. Infect. Public Health 14, 1750–1766. doi: 10.1016/j.jiph.2021.10.020, PMID: 34756812

[ref118] Uneputty A. Dávila-Lezama A. Garibo D. Oknianska A. Bogdanchikova N. Hernández-Sánchez J.. (2022). Strategies applied to modify structured and smooth surfaces: a step closer to reduce bacterial adhesion and biofilm formation. Colloid Interface Sci Commun 46:100560. doi: 10.1016/j.colcom.2021.100560

[ref119] Wang L. Hu C. Shao L. (2017). The antimicrobial activity of nanoparticles: present situation and prospects for the future. Int. J. Nanomedicine 12, 1227–1249. doi: 10.2147/IJN.S121956, PMID: 28243086 PMC5317269

[ref120] Wang B. Kuramitsu H. K. (2005). Inducible antisense Rna expression in the characterization of gene functions in *Streptococcus mutans*. Infect. Immun. 73, 3568–3576. doi: 10.1128/IAI.73.6.3568-3576.2005, PMID: 15908386 PMC1111864

[ref121] Wang J. Li J. Guo G. Wang Q. Tang J. Zhao Y.. (2016). Silver-nanoparticles-modified biomaterial surface resistant to staphylococcus: new insight into the antimicrobial action of silver. Sci. Rep. 6:32699. doi: 10.1038/srep32699, PMID: 27599568 PMC5013400

[ref122] Webber M. Piddock L. (2003). The importance of efflux pumps in bacterial antibiotic resistance. J. Antimicrob. Chemother. 51, 9–11. doi: 10.1093/jac/dkg050 12493781

[ref123] Weng L. Wu L. Guo R. Ye J. Liang W. Wu W.. (2022). Lactobacillus cell envelope-coated nanoparticles for antibiotic delivery against cariogenic biofilm and dental caries. J Nanobiotechnology 20:356. doi: 10.1186/s12951-022-01563-x, PMID: 35918726 PMC9344742

[ref124] Wu S. Liu Y. Zhang H. Lei L. (2019). Nano-graphene oxide improved the antibacterial property of antisense yycG Rna on *Staphylococcus aureus*. J. Orthop. Surg. Res. 14:305. doi: 10.1186/s13018-019-1356-x 31492154 PMC6731568

[ref125] Xu J. Li Y. Wang H. Zhu M. Feng W. Liang G. (2021). Enhanced antibacterial and anti-biofilm activities of antimicrobial peptides modified silver nanoparticles. Int. J. Nanomedicine 16, 4831–4846. doi: 10.2147/IJN.S315839, PMID: 34295158 PMC8291838

[ref126] Xu Y. Wang C. Hou J. Wang P. You G. Miao L. (2018). Mechanistic understanding of cerium oxide nanoparticle-mediated biofilm formation in *Pseudomonas aeruginosa*. Environ. Sci. Pollut. Res. Int. 25, 34765–34776. doi: 10.1007/s11356-018-3418-8, PMID: 30324376

[ref127] Yan X. He B. Liu L. Qu G. Shi J. Hu L.. (2018). Antibacterial mechanism of silver nanoparticles in *Pseudomonas aeruginosa*: proteomics approach. Metallomics 10, 557–564. doi: 10.1039/C7MT00328E, PMID: 29637212

[ref128] Yoshioka K. Kunieda T. Asami Y. Guo H. Miyata H. Yoshida-Tanaka K.. (2019). Highly efficient silencing of microrna by heteroduplex oligonucleotides. Nucleic Acids Res. 47, 7321–7332. doi: 10.1093/nar/gkz492, PMID: 31214713 PMC6698647

[ref129] You Y.-O. (2019). Virulence genes of Streptococcus mutans and dental caries. Int. J. Oral Biol. 44, 31–36. doi: 10.11620/IJOB.2019.44.2.31

[ref130] Younis A. B. Haddad Y. Kosaristanova L. Smerkova K. (2023). Titanium dioxide nanoparticles: Recent progress in antimicrobial applications. Wiley Interdiscip. Rev. Nanomed. Nanobiotechnol. 15:e1860. doi: 10.1002/wnan.1860, PMID: 36205103

[ref131] Zahmatkesh H. Mirpour M. Zamani H. Rasti B. (2023). Effect of samarium oxide nanoparticles fabricated by curcumin on efflux pump and virulence genes expression in Mdr Pseudomonas aeruginosa and *Staphylococcus aureus*. J. Clust. Sci. 34, 1227–1235. doi: 10.1007/s10876-022-02274-x

[ref132] Zhang J. Poh C. L. (2018). Regulating exopolysaccharide gene wcaF allows control of *Escherichia coli* biofilm formation. Sci. Rep. 8:13127. doi: 10.1038/s41598-018-31161-7, PMID: 30177768 PMC6120894

[ref133] Zhang Y. Sun P. Zhang L. Wang Z. Wang F. Dong K.. (2019). Silver-infused porphyrinic metal–organic framework: surface-adaptive, on-demand nanoplatform for synergistic bacteria killing and wound disinfection. Adv. Funct. Mater. 29:1808594. doi: 10.1002/adfm.201808594

[ref134] Zhao L. Ashraf M. (2015). Influence of silver-hydroxyapatite nanocomposite coating on biofilm formation of joint prosthesis and its mechanism. West Indian Med. J. 64, 506–513. doi: 10.7727/wimj.2016.179 27400164 PMC4961339

